# Reaching the end of the line: Operational issues with implementing phone-based unannounced pill counts in resource-limited settings

**DOI:** 10.1371/journal.pone.0185549

**Published:** 2017-10-19

**Authors:** Yael Hirsch-Moverman, Camilla Burkot, Suzue Saito, Koen Frederix, Blanche Pitt, Zenebe Melaku, Tsigereda Gadisa, Andrea A. Howard

**Affiliations:** 1 ICAP, Mailman School of Public Health, Columbia University, New York, NY, United States of America; 2 Department of Epidemiology, Mailman School of Public Health, Columbia University, New York, NY, United States of America; 3 Department of Sociomedical Sciences, Mailman School of Public Health, Columbia University, New York, NY, United States of America; University of Washington, UNITED STATES

## Abstract

**Introduction:**

Accurate measurement of adherence is necessary to ensure that therapeutic outcomes can be attributed to the recommended treatment. Phone-based unannounced pill counts were shown to be feasible and reliable measures of adherence in developed settings; and have been further used as part of medication adherence interventions. However, it is not clear whether this method can be implemented successfully in resource-limited settings, where cellular network and mobile phone coverage may be low. Our objective is to describe operational issues surrounding the use of phone-based unannounced pill counts in Lesotho and Ethiopia.

**Methods:**

Phone-based monthly unannounced pill counts, using an adaptation of a standardized protocol from previous US-based studies, were utilized to measure anti-TB and antiretroviral medication adherence in two implementation science studies in resource-limited settings, START (Lesotho) and ENRICH (Ethiopia).

**Results:**

In START, 19.6% of calls were completed, with 71.9% of participants reached at least once; majority of failed call attempts were due to phones not being available (54.8%) or because participants were away from the pills (32.7%). In ENRICH, 33.5% of calls were completed, with 86.7% of participants reached at least once; the main reasons for failed call attempts were phones being switched off (31.5%), participants not answering (27.3%), participants’ discomfort speaking on the phone (15.4%), and network problems (13.2%). Structural, facility-level, participant-level, and data collection challenges were encountered in these settings.

**Discussion:**

Phone-based unannounced pill counts were found to be challenging, and response rates suboptimal. While some of these challenges were specific to local contexts, most of them are generalizable to resource-limited settings. In a research study context, a possible solution to ease operational challenges may be to focus phone-based unannounced pill count efforts on a randomly selected sample from participants who are provided with study phones and rigorously ensure that call attempts are made for these participants.

## Introduction

It is well-recognized that the promise of efficacious therapies to treat and prevent long-term conditions cannot be met unless patients consistently adhere to prescribed regimens [1]. Medication adherence influences individual health outcomes, the cost of healthcare and, in the case of communicable infections, can influence transmission and the emergence of resistant strains. In clinical settings, accurate measurement of adherence is necessary to inform interpretation of treatment response and identify individuals in need of enhanced adherence support. In research settings, accurate measurement of adherence is necessary to ensure that therapeutic outcomes can be attributed to the assigned treatment, especially when an intervention effect is determined based on adherence. Unfortunately, accurate adherence measurement and monitoring remain significant challenges in both clinical and research settings.

In lieu of a single “gold standard” for measuring adherence [1], several *direct* and *indirect* measurement methods were developed. The ideal methods for measuring adherence vary depending on the setting and purpose of measuring adherence [2, 3]. Direct methods are generally more objective, yielding more reliable adherence assessments, but all methods have limitations. Electronic monitoring devices have been recommended to collect detailed adherence information but their use is limited by cost and risk of malfunction [3]. Pill counting is considered more feasible, and when used to measure antiretroviral adherence, it has been associated with viral suppression [4–7]. Some studies, however, failed to demonstrate an association between pill counts and viral load change [8]. Inaccuracies in clinic-based pill counts primarily stem from patients failing to bring their pills to the clinic, as well as emptying pill containers ("pill dumping”) prior to the appointment [9]. Bangsberg et al developed a home-based unannounced pill counting procedure, which reduces the opportunity to “pill dump”, because patients do not know the time of pill counts in advance [10]. Home-based unannounced pill counts virtually eliminate the potential problems faced by clinic-based pill counts, correlate with electronic medication monitoring [11, 12], and predict changes in viral load over time [10, 13].

Logistics and cost are major limitations of home-based unannounced pill counts. Unannounced home visits are often met with unanswered doors and concerns of privacy and confidentiality due to stigma. Additionally, these visits are logistically challenging and time-consuming in rural areas. To increase utility and reduce costs, Kalichman and colleagues adapted and tested a phone-based unannounced pill count protocol [14]. Phone-based unannounced pill counts were shown to be feasible and reliable measures of adherence in developed settings [14–18]; and have been further used as part of medication adherence interventions [19]. However, it is not clear whether this method can be implemented successfully in resource-limited settings, where cellular network and mobile phone coverage may be low. As part of the START (**S**tart **T**B patients on **A**RT and **R**etain on **T**reatment) and ENRICH (**EN**hance initiation and **R**etention in **I**PT **C**are for **H**IV) implementation science studies, we employed phone-based monthly unannounced pill counts to measure anti-tuberculosis (TB) and antiretroviral medication adherence. Our objective is to describe operational issues surrounding the use of phone-based unannounced pill counts in resource-limited settings.

## Materials and methods

START and ENRICH methods, including settings and facility characteristics, have been described elsewhere [20–22]. In brief, the START Study (ClinicalTrials.gov NCT01872390) is a cluster-randomized trial which evaluated the effectiveness, cost-effectiveness and acceptability of a combination intervention package (CIP) versus standard of care (SOC), to improve early antiretroviral therapy (ART) initiation, retention, and TB treatment success, among HIV-positive TB patients at 12 health facilities in Berea District, Lesotho, a nation with an HIV prevalence of 25% [23] and a TB incidence rate of 788 per 100,000 [24]. The ENRICH Study (ClinicalTrials.gov NCT01926379) is a cluster-randomized trial which evaluated the effectiveness of a CIP vs. SOC, to improve isoniazid preventive therapy (IPT) initiation, adherence, and completion among HIV-positive patients newly enrolled in HIV care at 10 health centers in Dire Dawa and Harari, Ethiopia, a nation with close to 800,000 people with HIV [25] and a TB incidence rate of 192 per 100,000 [24]. The follow-up period in both studies was six months.

Research Assistants (RA) received training on the unannounced pill count procedure and were provided with study phones to implement the pill counts. In both studies, monthly phone-based unannounced pill counts were conducted with all participants in both study arms. Pill counts were completed using an adaptation of a standardized protocol from previous studies [14], whereby participants were called at undisclosed times between monthly visits and asked to count their pills (S1 and S2 Files). Participants were not compensated for the pill count calls. At the baseline study visit, participants were trained to count their medications after answering the phone. In advance of the call, the RA recorded the name, dispense date, number of pills dispensed, number of doses per day, and number of pills per dose for each medication. Before proceeding with the pill counts, the RA first confirmed participants were in a private area and had access to their pills. The RA recorded the date, whether pills were taken before the call, and for each medication, how many pills were left. If a participant did not answer the call, the RA did not leave a message and attempted to reach the participant until the pill counts were completed, or five attempts have been made within the same month without answer or pill counts completion. The original study protocols called for three call attempts, but that was determined to be insufficient and after 15 and 12 months follow-up for START and ENRICH respectively, the number of attempts was increased to five. If the participant requested that the RA call back at another time, the RA did not schedule the callback at a specific time in order to maintain the pill count as ‘unannounced’.

We systematically collected quantitative and qualitative data on reasons for pill counts non-completion from study records, through weekly pill count completion reports submitted by RA (including attempt date, outcome, and if unsuccessful, the reason why), and conversations between RA and participants during study visits. Reasons for non-completed counts were recorded as open-ended responses that were later coded and categorized by the study investigators as network problems, numeracy issues, not having a phone, lack of privacy for the call, phone answered by someone other than the study participant, or not having pills at the time of the call. Qualitative data were analyzed using Microsoft Excel. Quantitative analyses were conducted using SAS 9.4 (SAS Institute Inc., Cary, NC). Participant characteristics and call outcomes were summarized with descriptive statistics using means, medians, proportions, and Pearson’s χ^2^.

In Lesotho, mobile phone ownership was high (86 and 102 mobile cellular subscriptions per 100 people in 2013 and 2014 respectively [26]); therefore, we did not distribute mobile phones to participants. However in Ethiopia, mobile phone ownership was much lower (27 and 32 per 100 people in 2013 and 2014, respectively [26]), and as the ENRICH study intervention included provision of adherence support messages to patients on IPT using interactive voice response technology, we distributed inexpensive mobile phones to participants in the CIP arm. Participants received a study-issued mobile phone, SIM card, and six months’ airtime vouchers at an approximate total cost of USD 45 per patient. Landlines in the home are practically nonexistent in the study locations, thus mobile phones were used exclusively to reach participants for pill counts.

All procedures performed in these studies were in accordance with the ethical standards of the institutional and/or national research committee and with the 1964 Helsinki Declaration and its later amendments or comparable ethical standards. The START Study was approved by the Columbia University Medical Center Institutional Review Board (CUMC-IRB) (Ref #IRB-AAAK7103) and Lesotho’s National Health Institutional Review Board and Ethics Research Committee (Ref #ID68-2012). The ENRICH Study was approved by the CUMC-IRB (Ref #IRB-AAAK3163) and the National Research Ethics Review Committee in Ethiopia (Ref #3.10/780/06). Informed consent was obtained from all individual participants included in the studies. Participants were informed that monthly phone-based unannounced pill counts will be conducted as part of the informed consent process.

## Results

START enrolled 371 participants, who were followed up for a median of 4.8 months; 2831 calls to 334 participants were attempted and 554 (19.6%) calls were completed. Attempts were not made for 37 (10%) participants who didn’t have a phone (n = 25) or could be contacted after hours only (n = 12). Participant characteristics are shown in Table 1. Overall, 240/334 (71.9%) START participants were reached at least once. The mean (±standard deviation [SD]) number of attempts per participant each month was 1.9±1.0. Completeness of calls was slightly higher during months 1 and 2 of follow-up at 24.8% and 25.6%, respectively; calls attempted during months 3–6 were completed 14.6%-17.9% of the time. Success rates were similar across study arms (21.0% vs. 18.8%, p = 0.17) and significantly higher for females than males (23.2% vs. 16.6%, p<0.001).

**Table 1:** Participant characteristics at baseline.

	START	ENRICH
	N = 371^a^	N = 316^b^
Age, median (IQR)	35 (30–44)	30 (26–38.5)
Female, n (%)	160 (43.1%)	200 (63.3%)
Marital status, n (%)		
- Married/living together	202 (54.4%)	130 (41.1%)
- Divorced/widowed	98 (26.4%)	131 (41.5%)
- Never married/lived together	71 (19.1%)	55 (17.4%)
Head of household (sole/shared), n (%)	168 (46.0%)	237 (75.0%)
Education, n (%)		
- None	29 (7.8%)	69 (21.8%)
- Primary	228 (61.5%)	159 (50.3%)
- Secondary or higher	114 (30.7%)	88 (27.9%)
Electricity in the home, n (%)	178 (48.0%)	239 (94.0%)
Own cell phone, n (%)	323 (87.1%)	220 (69.8%)

^a^Column numbers may not sum to totals due to missing data. Percentages are provided among non-missing data (n missing: age, 1; head of household, 6).

^b^Column numbers may not sum to totals due to missing data. Percentages are provided among non-missing data (n missing: own cell phone, 1).

ENRICH enrolled 316 participants, who were followed up for a median of 4.7 months; 3156 calls to 309 participants were attempted and 1058 (33.5%) calls were completed. Attempts were not made for 7 (2%) participants in the SOC study arm who didn’t have a phone (Table 1). Overall, 268/309 (86.7%) ENRICH participants were reached at least once. The mean (±SD) number of attempts per participant for each month was 2.1±1.4. Completeness of monthly calls was fairly consistent but declined from 36.6% in month 1 to 30.2% in month 6. Success rates were higher at CIP than SOC sites (38.4% vs. 27.2%, p<0.001); reasons differed by arm (p<0.001). SOC sites had a higher proportion of failed calls recorded as resulting from the patient having no phone (8.4% vs 1.5%, p < .0001) or being due to network problems (19.0% vs 7.7%, p < .0001). The most common reason for failure in the CIP arm was the phone was switched off (32.9%) followed by the phone was not answered (31.6%). Success rates did not differ between male and female participants.

As shown in Table 2, the majority of failed call attempts in START were due to the phone not being available (54.8%) or because the participant was away from the pills (32.7%). In ENRICH, the main reasons for failed call attempts were phone being switched off (31.5%), participants not answering the phone (27.3%), or participants’ discomfort with speaking on the phone (15.4%). Network problem was listed by the RA as the cause of failed calls 13.2% of the time.

**Table 2:** Reasons for failed calls.

	START	ENRICH
	N (%)	N (%)
Phone not available	1038 (54.2%)	0 (0%)
Network problem	12 (0.6%)	262 (13.2%)
Participant has no phone	0 (0%)	97 (4.9%)
Phone switched off	0 (0%)	624 (31.5%)
Phone unanswered	215 (11.2%)	541 (27.3%)
Participant uncomfortable to talk	0 (0%)	304 (15.4%)
Phone picked up by someone else	20 (1.0%)	90 (4.5%)
Participant away from the pills	626 (32.7%)	0 (0%)
Other problem	5 (0.3%)	61 (3.1%)

**Fig 1:**
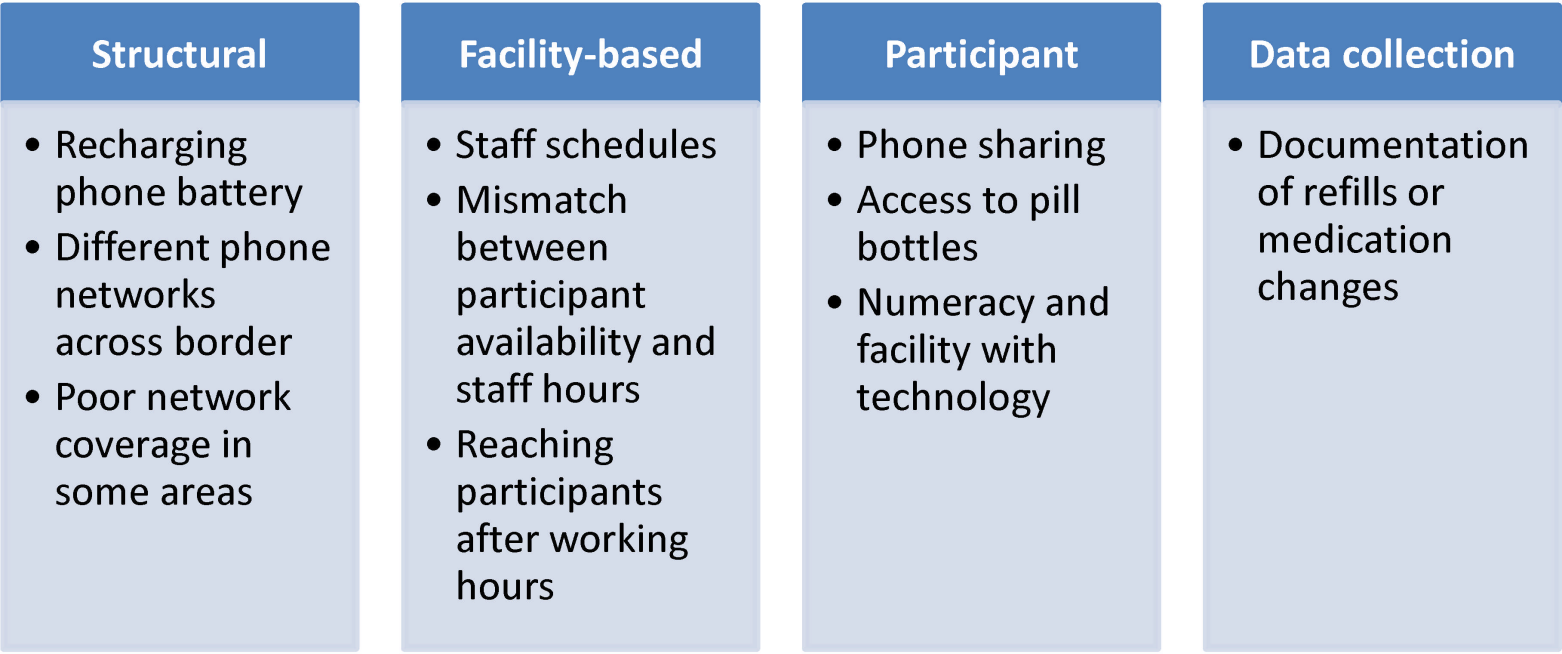
Implementation challenges of phone-based unannounced pill counts in resource-limited settings.

Many challenges were encountered in implementation of phone-based unannounced pill counts in these settings (Fig 1). In response, the respective study teams explored reasons for these challenges through conversations with participants at monthly study visits, and developed solutions as challenges arose over the course of the studies.

### Structural challenges

1. High phone *ownership* did not necessarily translate to high phone *availability*. Many participants ran out of battery or kept phones turned off intentionally to preserve battery because they were unable to recharge their phone regularly due to frequent power outages.
2. In Lesotho many participants worked in neighboring South Africa or lived near the border where network coverage is supplied by South African companies. As such, these participants often maintained a South African SIM card in their phone and were unreachable when study staff called their Lesotho number.
3. There was poor network coverage within some areas of Lesotho and Ethiopia.

### Attempted solutions

1. Participants were encouraged to bring their phones to the clinic so that they could charge the battery during their study visit, but the battery did not necessarily last until the next attempted unannounced pill count.
2. Participants were asked for their South African numbers and pill count attempts were made using a prepaid mobile phone equipped to make international calls.
3. Study staff varied the times of day at which they attempted pill counts to increase the likelihood of catching a participant when s/he might be traveling through or working in an area with more reliable coverage, as well as asking the participant whether s/he might be reached on other numbers.

### Facility-level challenges

1. Although making calls on a variable basis is critical for increasing the validity of the counts, it prevented staff from establishing a routine schedule.
2. Mismatch between participant availability and staff working hours, as some clinics closed early afternoon, severely restricting access to study records to prepare for pill counts.
3. Some participants worked very long hours, and could not be reached even during extended office hours. To protect participants' confidentiality and ensure data integrity, research staff could not remove study documents from the site, and thus could not make calls in the evening or on the weekends.

### Attempted solutions

1. In Lesotho, RA maintained a calendar to record pill count attempts, which assisted them in tracking pill count completion and outstanding attempts, and assisted the Study Coordinator in monitoring and ensuring call attempts were made. In Ethiopia, a module in the study database reminded RA when calls were due, and enabled them to log attempts.
2. The study team introduced some flexibility for study staff by staggering schedules and allowing study calls to be made from the head office rather than study clinics.
3. Arrangements were made for conducting pill counts from home using a separate sheet of paper and then transferring the information to the pill count forms in the office. Staff were trained to safeguard against potential violations of confidentiality, and were instructed to record only the minimum data required (i.e., participant name and phone number), to record no study identifiers, and to destroy the take-home paper after transferring the information to the study records.

### Participant-level challenges

1. Many participants shared phones with other household members.
2. Some participants were at work and did not have access to pill bottles or did not feel free to count them at work, due to the pervasive stigma.
3. While numeracy and facility with technology did not prove problematic in Lesotho, which has an adult literacy rate of 90% and high cell phone ownership, these did present significant challenges in Ethiopia, where only 39% of the adult population is literate [27]. Some participants were unable to count their pills independently and some lacked confidence in using their phones, including entering their passcode to unlock the phone.

### Attempted solutions

1. The number of attempts was increased from three to five so that participants who shared phones or were unable to count their pills at work were more likely to be reached.
2. Study staff were reminded to vary the times of day for pill count calls and to ask whether there was a preferred period of time when they would generally be available for pill counts.
3. Study staff assisted participants with simplifying their mobile phone passcode and practiced phone use and counting at study visits. If participants struggled to count when staff reached them on the phone, RA asked whether there was someone who could assist.

### Data collection challenges

Even if participants were reached and pill counts completed, challenges with knowing if a participant refilled or changed medications between visits remained, as clinic staff occasionally altered participants’ regimens between study visits in response to side effects or gave a larger supply of pills prior to travelling for an extended period.

### Attempted solutions

Study staff encouraged participants to inform them whenever they came to the clinic at an unscheduled time, continued reminding clinic staff about the study, and attempted to remain visible and approachable in the clinics.

## Discussion

To our knowledge, this is the first examination of the implementation of phone-based unannounced pill counts in resource-limited settings. In our implementation science studies in Lesotho and Ethiopia, phone-based unannounced pill counts were found to be challenging, with response rates varying between 20% and 34% in the two studies. Structural, facility-level, participant-level, and data collection challenges were encountered. While some challenges were specific to the local context (e.g., migration between Lesotho and South Africa; low cell phone ownership and literacy rates in Ethiopia), many are generalizable to resource-limited settings. It is important to note that the main contributors to failed calls were availability of phones, switched-off phones, and participants being away from the medicines. Many participants required as many as five calls to reach them, and many attempts did not result in successful completion of pill counts. It is important to consider the possibility that participants did not answer the phone when they recognized the study phone number because they could not, or did not want to, perform the pill count. This may have also contributed to the declining pill counts response rate over time. However, in these settings it’s not possible to have the caller number displayed as ‘private caller’.

While phone-based pill counts can be conducted in urban and rural areas, and missed phone contacts are inexpensive and allow staff to contact another participant without delay, they are also staff-intensive. It is crucial to carefully consider the practicalities of pill counts logistics, including the feasibility of making calls outside of normal work hours, and scheduling and ensuring attempts are actually made. In an effort to minimize implementation challenges, we consulted with experienced researchers who have used this procedure, albeit in developed settings, before study commencement. In a research study context, a possible solution to ease operational challenges may be to focus phone-based unannounced pill count efforts on a randomly selected sample from participants who are provided with study phones and rigorously ensure that up to five call attempts are made for these participants if they are not reached sooner. This approach may mitigate the potential for possible Hawthorne effects in two ways. First, the pill counts are unannounced and therefore participants do not know when they might be contacted; and second, participants will be randomly selected and therefore not know whether or not they will be contacted.

Earlier research experiences in the US found phone-based unannounced pill counts to be a feasible and reliable measure of medication adherence [14–17]. Among the greatest differences between early experiences and ours are structural challenges, including lack of mobile phone ownership, poor network availability, and participant mobility. Kalichman et al provided each participant with a dedicated mobile phone to maximize reachability [14]. Eligibility criteria in the study by Thompson et al included having a functioning phone [16]. As noted above, mobile phone ownership was very high in Lesotho and we supplied mobile phones to CIP participants in Ethiopia. Therefore, while phone ownership in our studies (except for SOC participants in Ethiopia) should have been comparable to prior studies, we suspect that unreliable network coverage and limited availability of electricity to charge phone batteries significantly impacted our participants’ ability to respond to calls. Kalichman et al found that 10/99 (10.1%) participants were unreachable by any phone, including the dedicated study phone given to them [15], compared to 94/334 (28.1%) of START participants and 41/309 (13.3%) of ENRICH participants. In a recent US study where participants were not provided with a mobile phone, Fredericksen et al found high concordance for phone-based and home-based unannounced pill counts [17]. The distribution of study mobile phones to CIP participants in Ethiopia had the potential to facilitate phone-based unannounced pill counts; however, phone use remained challenging in this setting. Surprisingly, when we compared response rates for study participants in the SOC arms, i.e. participants who did not receive a study phone, we found the response rate in Ethiopia to be higher than in Lesotho, which may be further indication that phone ownership does not guarantee good response. The relatively high level of population mobility in Lesotho, particularly among Basotho men, also posed a unique impediment to the completion of pill counts, which may account for a significantly higher response rate among females than males in Lesotho.

With respect to scheduling and management of the call attempts, we followed Kalichman [14] in having RA complete a baseline assessment with participants indicating the days and times which were most and least preferred to receive calls. Despite efforts to maximize the likelihood of successful calls by calling during acceptable times, the number of attempts required to reach our participants were similar to studies in developed settings. Kalichman et al reported that 57% of participants were reached on the first attempt, 23% on the second attempt, and 20% required ≥3 attempts [14], while Thompson et al found that nearly half of participants were contacted on the first attempt, with “the majority” being contacted by the second attempt [16]. These studies do not indicate common reasons why participants were not always reachable on the first or second attempt. Perhaps most notable is Fredericksen et al’s report that approximately four attempts were made to call participants before successfully reaching them [17]. Given the additional logistical and structural barriers present in resource-limited settings, the initial protocol of three attempts was perhaps overly optimistic. Authors of earlier studies do not provide details regarding human resources and call scheduling (e.g., whether a single staff member was dedicated to making calls, whether calls were attempted outside working hours), both of which would seem likely to increase pill count completion rates, but neither of which are likely to be feasible within public health systems in resource-limited settings. Integrating special software that helps manage the scheduling of these calls, such as maintaining a log of previous attempts and prompting additional attempts at given times, may have helped with their implementation. Having staff dedicated exclusively to the task of conducting unannounced pill counts might have alleviated some management and scheduling challenges, but such arrangements would be difficult to integrate into the structure of clinics in any future transition of implementation from research to clinic staff.

Participant confidence and competence in completing pill counts also offers a point of comparison. Kalichman et al report no participant was unable to count or required assistance, although they acknowledged this does not preclude the possibility of miscalculation or misidentification of medications [15]. This caveat is particularly important to bear in mind for populations with comorbidities, who are taking multiple medications. The major challenge reported by Kalichman and colleagues was that of participants struggling to manage and organize the pill count procedure, and they suggested repeated training and rehearsal with participants to minimize this problem; this additional training was undertaken in our studies by RA, village health workers (Lesotho), and peer educators (Ethiopia) to assist participants with the use of phones and with counting. While our studies in Lesotho and Ethiopia found that participants with low numeracy were in fact able to complete counts (contrary to prior expectations [15]), ultimately larger structural and logistical barriers overshadowed the overall feasibility of phone-based unannounced pill counts.

## Conclusion

Phone-based unannounced pill counts have proven challenging in two implementation science studies in resource-limited settings, which may cast a shadow over their utility in assessing adherence in research studies. However, in a research study context, a sample-based ascertainment of adherence using phone-based unannounced pill counts may offer a feasible solution to collecting adherence data in these settings.

## Supporting information

S1 File START Pill count form. (PDF)

S2 File ENRICH Pill count form. (PDF)

S3 File START Pill count dataset. (XLSX)

S4 File ENRICH Pill count dataset. (XLSX)

## References

[1] World Health Organization. Adherence to Long-term Therapies: Evidence for Action. Geneva 2003.

[2] Chesney MA. The elusive gold standard. Future perspectives for HIV adherence assessment and intervention. Journal of acquired immune deficiency syndromes. 2006;43 Suppl 1:S149–55. 17133199 10.1097/01.qai.0000243112.91293.26

[3] Williams AB, Amico KR, Bova C, Womack JA. A proposal for quality standards for measuring medication adherence in research. AIDS Behav. 2013;17(1):284–97. doi: 10.1007/s10461-012-0172-7 22407465 10.1007/s10461-012-0172-7 PMC3434290

[4] Bisson GP, Rowh A, Weinstein R, Gaolathe T, Frank I, Gross R. Antiretroviral failure despite high levels of adherence: discordant adherence-response relationship in Botswana. Journal of acquired immune deficiency syndromes. 2008;49(1):107–10. doi: 10.1097/QAI.0b013e3181820141 18667926 10.1097/QAI.0b013e3181820141 PMC5065011

[5] Moss AR, Hahn JA, Perry S, Charlebois ED, Guzman D, Clark RA, et al Adherence to highly active antiretroviral therapy in the homeless population in San Francisco: a prospective study. Clinical infectious diseases: an official publication of the Infectious Diseases Society of America. 2004;39(8):1190–8. 15486844 10.1086/424008

[6] San Lio MM, Carbini R, Germano P, Guidotti G, Mancinelli S, Magid NA, et al Evaluating adherence to highly active antiretroviral therapy with use of pill counts and viral load measurement in the drug resources enhancement against AIDS and malnutrition program in Mozambique. Clinical infectious diseases: an official publication of the Infectious Diseases Society of America. 2008;46(10):1609–16. 18419498 10.1086/587659

[7] Weidle PJ, Wamai N, Solberg P, Liechty C, Sendagala S, Were W, et al Adherence to antiretroviral therapy in a home-based AIDS care programme in rural Uganda. Lancet. 2006;368(9547):1587–94. doi: 10.1016/S0140-6736(06)69118-6 17084759 10.1016/S0140-6736(06)69118-6

[8] McNabb J, Ross JW, Abriola K, Turley C, Nightingale CH, Nicolau DP. Adherence to highly active antiretroviral therapy predicts virologic outcome at an inner-city human immunodeficiency virus clinic. Clinical infectious diseases: an official publication of the Infectious Diseases Society of America. 2001;33(5):700–5. 11486292 10.1086/322590

[9] Rabkin J, Chesney M. Treatment adherence to HIV medi cations: The Archilles heel of the new therapeutics In: Ostrow D, Kalichman S, editors. Behavioral and Mental Health Impacts of New HIV Therapies. New York: Plenum Press; 1999.

[10] Giordano TP, Guzman D, Clark R, Charlebois ED, Bangsberg DR. Measuring adherence to antiretroviral therapy in a diverse population using a visual analogue scale. HIV clinical trials. 2004;5(2):74–9. doi: 10.1310/JFXH-G3X2-EYM6-D6UG 15116282 10.1310/JFXH-G3X2-EYM6-D6UG

[11] Bangsberg DR, Hecht FM, Charlebois ED, Chesney M, Moss A. Comparing objective measures of adherence to HIV antiretroviral therapy: electronic medication monitors and unannounced pill counts. AIDS Behav. 2001;5:275–81.

[12] Bangsberg DR, Hecht FM, Charlebois ED, Zolopa AR, Holodniy M, Sheiner L, et al Adherence to protease inhibitors, HIV-1 viral load, and development of drug resistance in an indigent population. Aids. 2000;14(4):357–66. 10770537 10.1097/00002030-200003100-00008

[13] Oyugi JH, Byakika-Tusiime J, Charlebois ED, Kityo C, Mugerwa R, Mugyenyi P, et al Multiple validated measures of adherence indicate high levels of adherence to generic HIV antiretroviral therapy in a resource-limited setting. Journal of acquired immune deficiency syndromes. 2004;36(5):1100–2. 15247564 10.1097/00126334-200408150-00014

[14] Kalichman SC, Amaral CM, Stearns H, White D, Flanagan J, Pope H, et al Adherence to antiretroviral therapy assessed by unannounced pill counts conducted by telephone. Journal of general internal medicine. 2007;22(7):1003–6. doi: 10.1007/s11606-007-0171-y 17390095 10.1007/s11606-007-0171-y PMC2219717

[15] Kalichman SC, Amaral CM, Cherry C, Flanagan J, Pope H, Eaton L, et al Monitoring medication adherence by unannounced pill counts conducted by telephone: reliability and criterion-related validity. HIV clinical trials. 2008;9(5):298–308. doi: 10.1310/hct0905-298 18977718 10.1310/hct0905-298 PMC2937191

[16] Thompson N, Nazir N, Cox LS, Faseru B, Goggin K, Ahluwalia JS, et al Unannounced telephone pill counts for assessing varenicline adherence in a pilot clinical trial. Patient preference and adherence. 2011;5:475–82. doi: 10.2147/PPA.S24023 22003285 10.2147/PPA.S24023 PMC3191924

[17] Fredericksen R, Feldman BJ, Brown T, Schmidt S, Crane PK, Harrington RD, et al Unannounced telephone-based pill counts: a valid and feasible method for monitoring adherence. AIDS Behav. 2014;18(12):2265–73. doi: 10.1007/s10461-014-0916-7 25331265 10.1007/s10461-014-0916-7 PMC4495998

[18] Kalichman SC, Cherry C, Kalichman MO, Amaral C, White D, Grebler T, et al Randomized clinical trial of HIV treatment adherence counseling interventions for people living with HIV and limited health literacy. Journal of acquired immune deficiency syndromes. 2013;63(1):42–50. doi: 10.1097/QAI.0b013e318286ce49 23337369 10.1097/QAI.0b013e318286ce49 PMC4279917

[19] Kalichman SC, Kalichman MO, Cherry C, Swetzes C, Amaral CM, White D, et al Brief behavioral self-regulation counseling for HIV treatment adherence delivered by cell phone: an initial test of concept trial. AIDS patient care and STDs. 2011;25(5):303–10. doi: 10.1089/apc.2010.0367 21457056 10.1089/apc.2010.0367 PMC3085946

[20] Howard AA, Hirsch-Moverman Y, Frederix K, Daftary A, Saito S, Gross T, et al The START Study to evaluate the effectiveness of a combination intervention package to enhance antiretroviral therapy uptake and retention during TB treatment among TB/HIV patients in Lesotho: rationale and design of a mixed-methods, cluster-randomized trial. Glob Health Action. 2016;9:31543. 10.3402/gha.v9.31543 PMC4926099 27357074

[21] O’Connor DE, Frederix K, Saito S, Maama LB, Hirsch-Moverman Y, Pitt B, et al Pulmonary Tuberculosis Diagnostic Practices among People Living with HIV in Lesotho. Int J Tuberc Lung Dis. Forthcoming 2017. 10.5588/ijtld.17.0297 PMC5652316 28911357

[22] Howard AA, Hirsch-Moverman Y, Saito S, Gadisa T, Daftary A, Melaku Z. The ENRICH Study to evaluate the effectiveness of a combination intervention package to improve isoniazid preventive therapy initiation, adherence and completion among people living with HIV in Ethiopia: rationale and design of a mixed methods cluster randomized trial. Contemp Clin Trials Commun. 2017;6:46–54. doi: 10.1016/j.conctc.2017.03.001 28626811 10.1016/j.conctc.2017.03.001 PMC5470840

[23] Lesotho Ministry of Health, ICF International. Lesotho Demographic and Health Survey 2014. Key Indicators Report. Maseru; 2015.

[24] World Health Organization. Global tuberculosis control 2015. Geneva; WHO 2015 http://www.who.int/tb/publications/global_report/gtbr15_main_text.pdf. Accessed Jan 20, 2016

[25] Federal HIV/AIDS Prevention and Control Office (FHAPCO). Country Progress Report on the HIV Response. 2014.

[26] The World Bank. Mobile cellular subscriptions (per 100 people) [Available from: http://data.worldbank.org/indicator/IT.CEL.SETS.P2.

[27] UN_UNESCO Institute for Statistics (UIS). Adult literacy rate [Available from: http://data.un.org/Data.aspx?d=SOWC&f=inID%3A74.

