# Supplementary material for: Reaching the end of the line: Operational issues with implementing phone-based unannounced pill counts in resource-limited settings
Source: PLoS One. 2017 Oct 19;12(10):e0185549. doi: 10.1371/journal.pone.0185549 (PMC5648117; doi:10.1371/journal.pone.0185549)
Supplement: S1 File — (PDF) [file pone.0185549.s001.pdf]

START Pill Count Form

SID: ____ -MC- ________

"I am calling you today so we can count together the number of HIV and TB pills that are left in your medication bottles/blister packs. Are you in a private area where you can discuss your pills?" ***(If NO- call at different time to complete form. If YES- continue). "*** **First, please tell me if you've taken your HIV and TB medication today"** ***(write answer of yes or no in column below)*** **. "Next, please take a moment to bring all of your medication bottles and blister packs to the phone so we can count together how many pills you have left."** ***(If patient cannot count, ask if there is someone else there who can help them count. If NO, do not complete parts of form requiring counting and indicate reason of 'participant cannot count.') Refer to Pill Count Facesheet for regimen and codes. If HIV regimen is dispensed in separate pills and bottles, separate them by HIV drug A, HIV drug B, and HIV drug C and use individual codes for each. If the patient is receiving HIV medications in combined dose/pills, then only enter on HIV drug A and include combined regimen code.***

HIV Medications

		Date patient reached by phone (dd/mm/yyyy)	Date drugs dispensed (dd/mm/yyyy)	Number of pills dispensed at last visit	Number of doses per day	Pills taken per dose	Number of days since pills dispensed	Did patient take pills today? (yes/no)	Number of pills left	Code of drug
Month 1	HIV drug A									
	HIV drug B									
	HIV drug C									
Month 2	HIV drug A									
	HIV drug B									
	HIV drug C									
Month 3	HIV drug A									
	HIV drug B									
	HIV drug C									
Month 4	HIV drug A									
	HIV drug B									
	HIV drug C									
Month 5	HIV drug A									
	HIV drug B									
	HIV drug C									
Month 6	HIV drug A									
	HIV drug B									
	HIV drug C									
Month 7	HIV drug A									
	HIV drug B									
	HIV drug C									
Month 8	HIV drug A									
	HIV drug B									
	HIV drug C									
Month 9	HIV drug A									
	HIV drug B									
	HIV drug C									
Month 10	HIV drug A									
	HIV drug B									
	HIV drug C									

ARV 1st line regimen (combined)	1st line HIV (combined) regimen or drug code	ARV 2nd line regimen (combined)	2nd line HIV (combined) regimen or drug code
TDF - 3TC - EFV	1a	AZT - 3TC - LPV/r	2a
TDF - 3TC - NVP	1b	TDF - 3TC - LPV/r	2b
AZT - 3TC - NVP	1c	AZT - 3TC - ATV/r	2c
AZT - 3TC - EFV	1d	TDF - 3TC - ATV/r	2d
ABC - 3TC - NVP	1e	DDI - ABC - LPV/r	2e
ABC - 3TC - EFV	1f	DDI - 3TC - LPV/r	2f
Other ARV regimen*	1g		

** For other ARV regimen, specify the drug next to the code above*

Individual HIV drugs (1st and 2nd line regimens)	Individual HIV drug code
TDF	3a
3TC	3b
EFV	3c
AZT	3d
NVP	3e
ABC	3f
LPV/r	3g
ATV/r	3h
DDI	3i
Other individual code*	3j

*** For other individual HIV drug code, specify drug next to the code above*

Columbia University IRB
IRB-AAAK7103
IRB Approval Date: 09/25/2014
for use until: 09/24/2015

START Pill Count Form

SID: ☐☐ -MC- ☐☐☐☐

TB Medications

	Date patient reached by phone (dd/mm/yyyy)	Date drugs dispensed (dd/mm/yyyy)	Number of pills dispensed at last visit	Number of doses per day	Pills taken per dose	Number of days since pills dispensed	Did patient take pills today? (yes/no)	Number of pills left	Code of drug
**Month 1**									
**Month 2**									
**Month 3**									
**Month 4**									
**Month 5**									
**Month 6**									
**Month 7**									
**Month 8**									
**Month 9**									
**Month 10**									

**TB drug code**	**TB Regimen or drug code**
RH	01
RHZES	02
RHZE	03
RHE	04
Other TB treatment regimen*	06

** For other TB treatment regimen, specify the drug next to the code above*

Columbia University IRB
IRB-AAAK7103
IRB Approval Date: 09/25/2014
for use until: 09/24/2015
