# Supplementary material for: Reaching the end of the line: Operational issues with implementing phone-based unannounced pill counts in resource-limited settings
Source: PLoS One. 2017 Oct 19;12(10):e0185549. doi: 10.1371/journal.pone.0185549 (PMC5648117; doi:10.1371/journal.pone.0185549)
Supplement: S2 File — (PDF) [file pone.0185549.s002.pdf]

ENRICH Pill Count Form

Study Identification Number ☐☐-MC- ☐☐ ☐☐

"I am calling you today so we can count together the number of HIV and IPT pills that are left in your medication bottles/plastic packs. Are you in a private area where you can discuss your pills?" (If NO- call at different time to complete form. If YES- continue). "First, please tell me if you've taken your HIV and IPT medication today." (write answer of yes or no in column below). "Next, please take a moment to bring your HIV and IPT medication bottles and plastic packs to the phone so we can count together how many pills you have left." (If patient cannot count, ask if there is someone else there who can help them count. If NO, do not complete parts of form requiring counting and indicate reason of 'participant cannot count.') *Refer to Pill Count Facesheet for dispensing date, number of pills dispensed, regimen and codes. Begin by asking about IPT pills and when completed, ask participant after HIV pills.For all dates, use Ethiopian calendar.*

IPT									
Month in the study	Date patient reached by phone (dd/mm/yyyy)	Date drugs dispensed (dd/mm/yyyy)	Number of pills dispensed at last visit	Number of doses per day	Pills taken per dose	Number of days since dispensation	Did patient take pills today? (yes/no)	Number of pills left	Code of drug
Month 1				1					
Month 2				1					
Month 3				1					
Month 4				1					
Month 5				1					
Month 6				1					
Month 7				1					
Month 8				1					
Month 9				1					
Month 10				1					

HIV									
Month in the study	Date patient reached by phone (dd/mm/yyyy)	Date drugs dispensed (dd/mm/yyyy)	Number of pills dispensed at last visit	Number of doses per day	Pills taken per dose	Number of days since dispensation	Did patient take pills today? (yes/no)	Number of pills left	Code of drug
Month 1									
Month 2									
Month 3									
Month 4									
Month 5									
Month 6									
Month 7									
Month 8									
Month 9									
Month 10									

ART 1st line regimen	Regimen code
d4 t- 3TC-NVP	1a
d4t - 3TC - EFV	1b
AZT - 3TC - NVP	1c
AZT - 3TC - EFV	1d
TDF - 3TC - EFV	1e
TDF - 3TC - NVP	1f
TDF - FTC - EFV	1g
ABC - 3TC - NVP	1h
ABC - 3TC - EFV	1i
ABC - 3TC - AZT	1j
Other, specify	1k

ART 2nd line regimen	Regimen code
ABC - ddi - LPV/r	2a
TDF - ddi - LPV/r	2b
TDF - 3TC - LPV/r	2c
AZT - 3TC - LP/r	2d
Other, specify	2e

IPT regimen	Regimen Code
INH	0
INH+B6	1
Other, specify	2

ENRICH Pill Count Facesheet

***Instructions* : Update this form during every study visit with MC participant. Information captured here should be referred to when completing unnannounced Pill Count Form.**

**Preferred Days of week for phone calls:**	
**Day(s) of week DOES NOT wish to receive calls:**	
**Preferred times of day for phone calls:**	
**Time(s) of day DOES not wish to receive calls:**	
**Preferred phone number to call:**	

Instructions: To complete for IPT, refer to pill bottle/plastic pack dispensed to participant at clinic visit.

IPT Pills

Date Dispensed (dd/mm/yyyy)	Regimen Dispensed (use codes below)	Total number of pills dispensed	Time of day doses taken

Instructions: To complete for ART, refer to pill bottle dispensed to participant at clinic visit.

ART Pills

Date Dispensed (dd/mm/yyyy)	Regimen Dispensed (use codes below)	Total number of pills dispensed	Time of day doses taken

ART 1st line regimen	Regimen code
d4 t- 3TC-NVP	1a
d4t - 3TC - EFV	1b
AZT - 3TC - NVP	1c
AZT - 3TC - EFV	1d
TDF - 3TC - EFV	1e
TDF - 3TC - NVP	1f
TDF - FTC - EFV	1g
ABC - 3TC - NVP	1h
ABC - 3TC - EFV	1i
ABC - 3TC - AZT	1j
Other, specify	1k

ART 2nd line regimen	Regimen code
ABC - ddi - LPV/r	2a
TDF - ddi - LPV/r	2b
TDF - 3TC - LPV/r	2c
AZT - 3TC - LP/r	2d
Other, specify	2e

IPT regimen	Regimen code
INH	0
INH+B6	1
Other, specify	2
